# Comparative Transcriptomic Analysis Identifies a Range of Immunologically Related Functional Elaborations of Lymph Node Associated Lymphatic and Blood Endothelial Cells

**DOI:** 10.3389/fimmu.2019.00816

**Published:** 2019-04-16

**Authors:** Stella J. Berendam, Alexander F. Koeppel, Nicole R. Godfrey, Sherin J. Rouhani, Amber N. Woods, Anthony B. Rodriguez, J. David Peske, Kara L. Cummings, Stephen D. Turner, Victor H. Engelhard

**Affiliations:** ^1^Department of Microbiology, Immunology, and Cancer Biology, Carter Immunology Center, University of Virginia School of Medicine, Charlottesville, VA, United States; ^2^Department of Public Health Sciences and Bioinformatics Core, University of Virginia School of Medicine, Charlottesville, VA, United States

**Keywords:** endothelial cell, lymph node, lymphatic, RNA-Seq, antigen presentation, scavenger receptors, chemokines, cytokines and receptors

## Abstract

Lymphatic and blood vessels are formed by specialized lymphatic endothelial cells (LEC) and blood endothelial cells (BEC), respectively. These endothelial populations not only form peripheral tissue vessels, but also critical supporting structures in secondary lymphoid organs, particularly the lymph node (LN). Lymph node LEC (LN-LEC) also have been shown to have important immunological functions that are not observed in LEC from tissue lymphatics. LN-LEC can maintain peripheral tolerance through direct presentation of self-antigen via MHC-I, leading to CD8 T cell deletion; and through transfer of self-antigen to dendritic cells for presentation via MHC-II, resulting in CD4 T cell anergy. LN-LEC also can capture and archive foreign antigens, transferring them to dendritic cells for maintenance of memory CD8 T cells. The molecular basis for these functional elaborations in LN-LEC remain largely unexplored, and it is also unclear whether blood endothelial cells in LN (LN-BEC) might express similar enhanced immunologic functionality. Here, we used RNA-Seq to compare the transcriptomic profiles of freshly isolated murine LEC and BEC from LN with one another and with freshly isolated LEC from the periphery (diaphragm). We show that LN-LEC, LN-BEC, and diaphragm LEC (D-LEC) are transcriptionally distinct from one another, demonstrating both lineage and tissue-specific functional specializations. Surprisingly, tissue microenvironment differences in gene expression profiles were more numerous than those determined by endothelial cell lineage specification. In this regard, both LN-localized endothelial cell populations show a variety of functional elaborations that suggest how they may function as antigen presenting cells, and also point to as yet unexplored roles in both positive and negative regulation of innate and adaptive immune responses. The present work has defined in depth gene expression differences that point to functional specializations of endothelial cell populations in different anatomical locations, but especially the LN. Beyond the analyses provided here, these data are a resource for future work to uncover mechanisms of endothelial cell functionality.

## Introduction

Lymphatic and blood vessels are formed by specialized endothelial cells that are closely related but distinct (1). These endothelial populations form vessels in peripheral tissue, but also supporting structures in secondary lymphoid organs, particularly lymph node (LN). Blood endothelial cells (BEC) form high endothelial venules, which control the entry of lymphocytes from the bloodstream, while lymphatic endothelial cells (LEC) form lymphatic sinuses that control entry of tissue-localized immune cells, and organization and exit of all immune cells, in addition to the flow of lymph. To determine the basis for these functional attributes, several studies have evaluated transcriptomes of LEC and BEC, primarily from peripheral tissue vessels. Most of these have used microarray approaches and often relied on endothelial cells cultured *in vitro* (1–11), (see also EndoDB (12) for a comprehensive listing of prior studies, associated databases, and analysis tools). While they have revealed differences in LEC and BEC in genes implicated in vascular tube formation, transport of solutes, and immune cell trafficking, microarray hybridization-based approaches posed several limitations, including high background levels and limited range of detection. Furthermore, these studies also concluded that even short-term primary cultures of LEC and BEC *ex vivo* resulted in some level of de-differentiation. Additionally, these studies used cells isolated from the skin and did not compare LEC and BEC from different anatomical sites. Analysis of transcriptional programs to understand the functionality and diversity of LEC and BEC in different anatomical locations remains to be done.

Recent studies have demonstrated that LN-associated LEC (LN-LEC) also actively participate in controlling innate and adaptive immune responses. We previously demonstrated that LN-LEC, but not LEC in tissue lymphatics, adventitiously expressed transcripts for proteins otherwise restricted to a small number of peripheral tissues. We showed that a peptide epitope from one of these, the melanocyte protein tyrosinase (Tyr), was presented on LN-LEC associated MHC-I molecules to Tyr-specific CD8 T cells (13–15). Although this induced activation and proliferation, LN-LEC also expressed high levels of PD-L1 that resulted in deletion of Tyr-specific CD8 T cells (15). LEC from tissue lymphatics express negligible levels of PD-L1 (14). In a separate study, we established that LN-LEC could induce Lag3 dependent CD8 T cell deletion via expression of MHC-II molecules, and that LEC from tissue lymphatics express negligible levels of MHC-II (16). While LN-LEC were incapable of presenting acquired Ag via these MHC-II molecules, they nonetheless transferred endogenous antigens to dendritic cells (DC) for presentation to CD4 T cells, resulting in anergy (16). These results point to an important role for LN-LEC in establishing systemic peripheral T cell tolerance. Conversely, others have shown that LN-LEC capture and archive exogenous antigens that induce antigen-specific memory CD8 T cell persistence (17). This occurs via transfer of LEC-archived antigens to migratory DC as a result of LEC apoptosis during LN contraction and also via direct exchange of archived antigens by the two cell types (18). The molecular mechanisms involved in these different processes of antigen acquisition, expression, and transfer by LN-LEC remain unclear, and the specific microenvironmental influences that control the phenotypic as well as functional distinctions between LEC in the LN and in the periphery remain to be fully understood.

In this study, we address these issues, as well as the technical limitations of previous studies, by using RNA-Seq analysis to compare the transcriptomes of freshly isolated murine LN-associated LEC and BEC (LN-BEC) as well as freshly isolated LEC from the diaphragm (D-LEC) as representative of peripheral tissue lymphatics. RNA-Seq has greatly improved the analysis of whole transcriptomes with higher sensitivity and dynamic range coupled to lower technical variations compared to microarrays and quantitative PCR (19, 20). Our work provides an important resource for further exploration of endothelial cell functionality in different anatomical locations.

## Results and Discussion

### LN-LEC, LN-BEC, and D-LEC Are Transcriptionally Distinct

LEC and BEC populations were purified from relevant tissues using magnetic bead enrichment and electronic cell sorting from 10 to 33 C57BL/6 mice for each replicate sample, and subjected to RNA-Seq (Figures S1A,B). This yielded 48–98 million reads per replicate, with an average length of 180 nucleotides, and an average of 85.7% uniquely mapped reads. These reads mapped a total of 23,284 genes. One previous study estimated that one transcript copy per liver cell corresponds to 3 FPKM (21), while another estimated that genes expressed at FPKM > 1 were reproducibly and accurately detected in bulk RNA-Seq experiments (22). Based on this, we identified genes with FPKM ≥ 1 and p-adjusted < 0.05 in all replicate comparisons, which ensures that low level FPKM values are consistent. This gave a total of 15,331 genes considered to be expressed in at least one cell population (Table S1). Similar gene numbers were expressed in LN-LEC, LN-BEC, and D-LEC, respectively (Figure S1C). Principal component analysis revealed that the transcriptional profiles of replicates clustered tightly, and LN-LEC, LN-BEC, and D-LEC differed from each other (Figure S2A). The pan-endothelial marker (CD31) was strongly expressed in all endothelial cell populations (Table 1). Established markers of LEC (LYVE-1, PDPN, PROX-1, and RELN) were strongly expressed in LN-LEC and D-LEC with minimal (1.0–3.4%) cross expression by LN-BEC (Table 1). Established markers of BEC (NRP-1, VEGFR-1, VWF, and NOTCH-4) were strongly expressed in LN-BEC with minimal cross expression in LN-LEC and D-LEC (0.2–4.7%). The low levels of cross expression of these genes are consistent with very low cross-contamination or genuine low-level expression. Known markers of fibroblast reticular cells and hematopoietic subpopulations were evident only at very low to negligible levels (Table 1). Consistent with our previous findings (13, 14), Tyr was expressed by LN-LEC but not LN-BEC and D-LEC, while (PD-L1) was expressed at high levels in LN-LEC and LN-BEC but not D-LEC. These data established a high level of confidence in further analyzing gene expression patterns of that differ among LN-LEC, LN-BEC, and D-LEC.

**Table 1:** RNA-seq validation of stromal cell-specific markers and hematopoietic cell-lineage markers based on normalized gene expression levels (FPKM).

**Lineage**	**Gene**	**Average LN-LEC**	**Average D-LEC**	**Average LN-BEC**
Endothelial	**CD31/Pecam1**	30662	31820	98007
Lymphatic endothelial	Pdpn	**18631**	**63207**	632
	Lyve1	**60105**	**165926**	1730
	Prox1	**8005**	**7925**	228
	Reln	**2994**	**85960**	78
Blood endothelial	Vegfr1 (Flt1)	195	61	**14897**
	Vwf	125	164	**6455**
	Notch4	92	198	**4188**
	Nrp1	666	106	**46414**
Fibroblastic reticular	Pdgfra	184	98	227
	Pdgfrb	90	35	345
	Des	96	49	161
Hematopoietic	Cd45	40	15	289
T cell	Cd3 (d,e,g)	6	3	168
	Cd8 (a,b1)	8	3	62
	Cd4	13	8	26
B cell	Cd19	18	4	183
	Cd20	17	6	125
Dendritic cell	Cd11c	3	2	4
Macrophage	Cd11b	1	3	7
Tolerogenic profile	Tyr	**346**	9	19
	Cd274	**11612**	866	**9798**
Housekeeping genes	Actb	1,052,300	1,561,806	1,328,475
	Hprt	3837	5214	4941

*Genes and FPKM values highlighted in bold represent previously identified lineage and phenotypic markers associated with the cell types*.

### Differential Gene Analyses Reveal Subsets of Genes Specific Only to LN-LEC, LN-BEC, or D-LEC, and Subsets of Genes Shared by at Least Two Cell Populations

**Figure 1:**
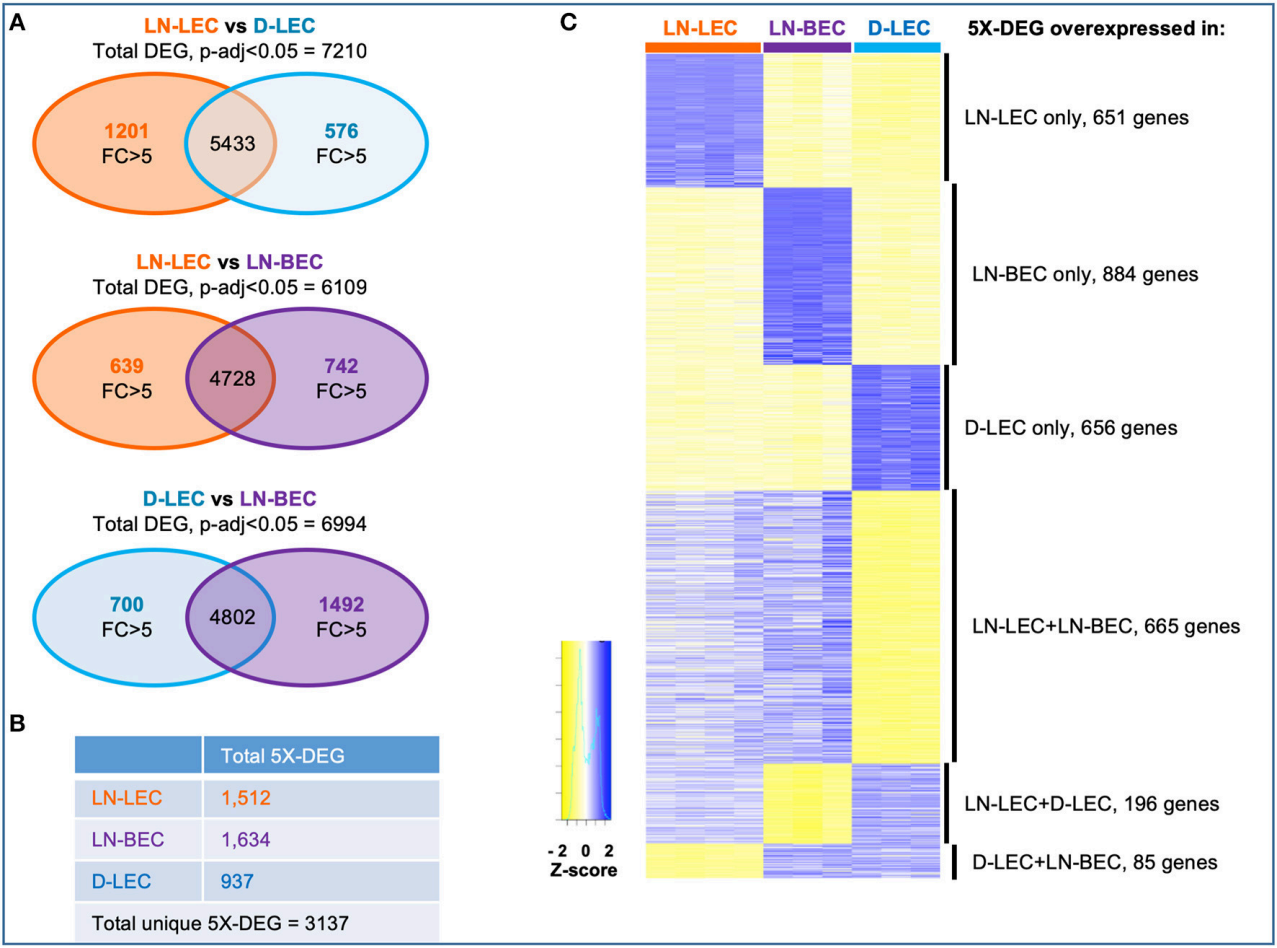
Differential gene analysis and hierarchical clustering of differentially expressed genes in LN-LEC, LN-BEC, and D-LEC revealed distinct and shared subsets by at least two populations. **(A)** Venn diagrams showing pairwise comparisons of LN-LEC, LN-BEC, and D-LEC. **(B)** Total number of unique and cell type specific 5X-DEG. **(C)** Hierarchical clustering of distinct and shared subsets of 5X-DEG. Complete lists of 5X-DEGs in each subset are listed in Table S2.

Genes that were differentially expressed between any two cell types (DEG) were identified based on an adjusted *p* < 0.05. Comparisons of LN-LEC vs. D-LEC, LN-LEC vs. LN-BEC, and D-LEC vs. LN-BEC identified 7210, 6109, and 6994 DEG, respectively (Figure 1A). We next identified genes whose differential expression in these pairwise comparisons was greater than 5-fold (5X-DEG). A total of 1512, 1634, and 937 5X-DEG were overexpressed in LN-LEC, LN-BEC, and D-LEC respectively, accounting for a total of 3137 unique 5X-DEG (Figures 1A,B). Since the total expressed genes in these populations were similar (Figure S1C), the substantially higher numbers of 5X-DEG in LN-BEC and LN-LEC relative to D-LEC suggests that the two LN populations have more elaborated functionalities.

Hierarchical clustering identified subsets of 5X-DEG distinct to only LN-LEC, LN-BEC, and D-LEC, and subsets shared by two cell types: LN-LEC+LN-BEC, LN-LEC+D-LEC, and D-LEC+LN-BEC (Figure 1C; Table S2). There were relatively few 5X-DEG in the D-LEC+LN-BEC shared subset, consistent with the distinct developmental origins and anatomical locations of these two populations. Intriguingly, the LN-LEC+LN-BEC shared subset contained 3.4 times more 5X-DEG than the LN-LEC+D-LEC shared subset. Thus, despite their different developmental origins, the two LN-localized endothelial populations are more transcriptionally related to one another than the two LEC populations that occupy distinct anatomical niches.

We used GOrilla software to identify biological process and molecular function Gene Ontology (GO) terms in each 5X-DEG subset that were highly ranked based on enrichment score (see Methods), which emphasizes co-expression of multiple genes associated with a term, rather than overexpression of individual genes. We identified GO terms with significant (p-adjusted < 0.001) enrichment scores in all 5X-DEG subsets except D-LEC+LN-BEC (Figure S2B), and this subset was thus excluded from the analyses below. These GO terms were often interrelated and were further grouped into clusters based on visual inspection (Table S3; Figure S2C). These clusters, and the overexpressed genes that they contained, are discussed in more details in sections below.

### Differential Expression of Extracellular Matrix Components and Cell Adhesion Molecules Suggest Specialized Structural and Functional Attributes of LEC and BEC in Distinct Tissue Microenvironments

**Figure 2:**
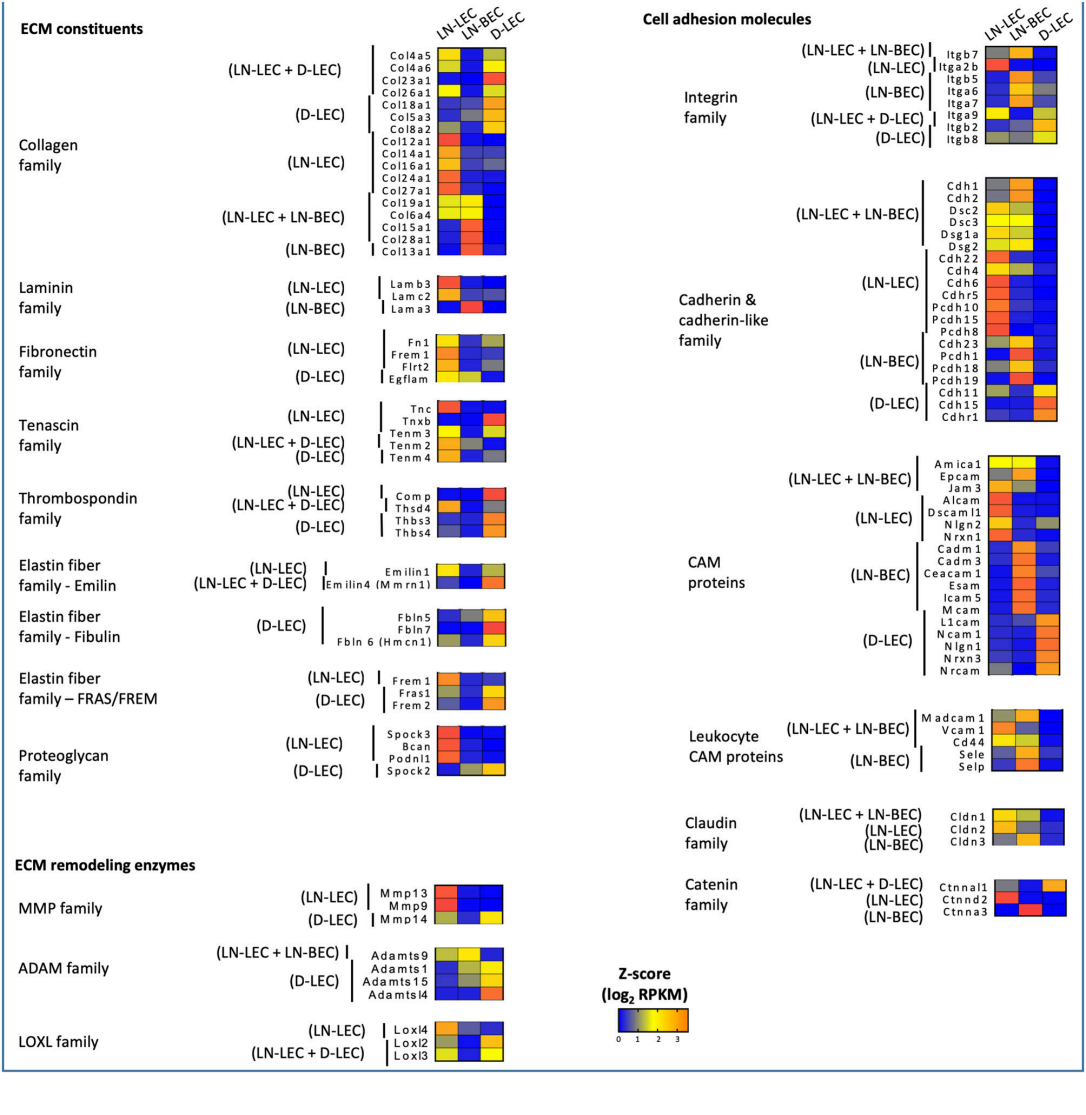
5X-DEG in all five subsets expressed distinct and shared members of the ECM constituents, remodeling enzymes, cell adhesion molecules. Heatmap analysis based on Z-score values of average log2 FPKM for replicates in each cell type (details in Methods).

GO terms related to extracellular matrix (ECM) were highly ranked in all 5 5X-DEG subsets and identified overexpressed genes in several different processes and functions. Each subset overexpressed different collagen molecules while 3 laminin family members were overexpressed in one of the two LN endothelial cell populations, and 4 fibronectin family members were overexpressed in one of the two LEC populations (Figure 2). Members of the tenascin, thrombospondin, elastin, and proteoglycan families were overexpressed in one or both of the two LEC populations, but none were overexpressed in LN-BEC. Similarly, ECM remodeling enzymes of the MMP, ADAM, and LOXL families were widely overexpressed in the LEC subpopulations, but minimally in LN-BEC. These data point to an elaboration of ECM components in LEC compared to BEC, and also suggest that LEC in different anatomical locations create distinct ECM microenvironments through both synthesis and remodeling activities. These may contribute to distinct structural and functional attributes of adjacent lumenal and ablumenal compartments.

GO terms related to cell adhesion were also highly ranked in all 5 5X-DEG subsets. Each of the 5 subsets overexpressed different integrin molecules (Figure 2). The LN-LEC, LN-BEC, D-LEC, and LN-LEC+LN-BEC also overexpressed different cadherin and cadherin-like family members and cell adhesion molecules (CAMs), known to mediate homophilic adhesion of endothelial population of common lineage and origin. No integrins or cadherin family members were overexpressed in the LN-LEC+D-LEC subset, suggesting a significant distinction in the patterns of cell engagement by these two LEC subpopulations. The leukocyte CAMs, all of which play well-established roles in mediating the extravasation of cells into lymphoid and peripheral tissues, were overexpressed in the LN-BEC only, but also LN-LEC+LN-BEC 5X-DEG subsets. Their function in LN-LEC remains to be established. Claudin and catenin family members were also overexpressed almost exclusively in the LN endothelial populations, with only a single catenin gene overexpressed in D-LEC associated subset. Taken together, these data suggest that LN endothelial subpopulations are endowed with an enriched capacity for interactions with a diversity of other cells relative to D-LEC.

**Figure 3:**
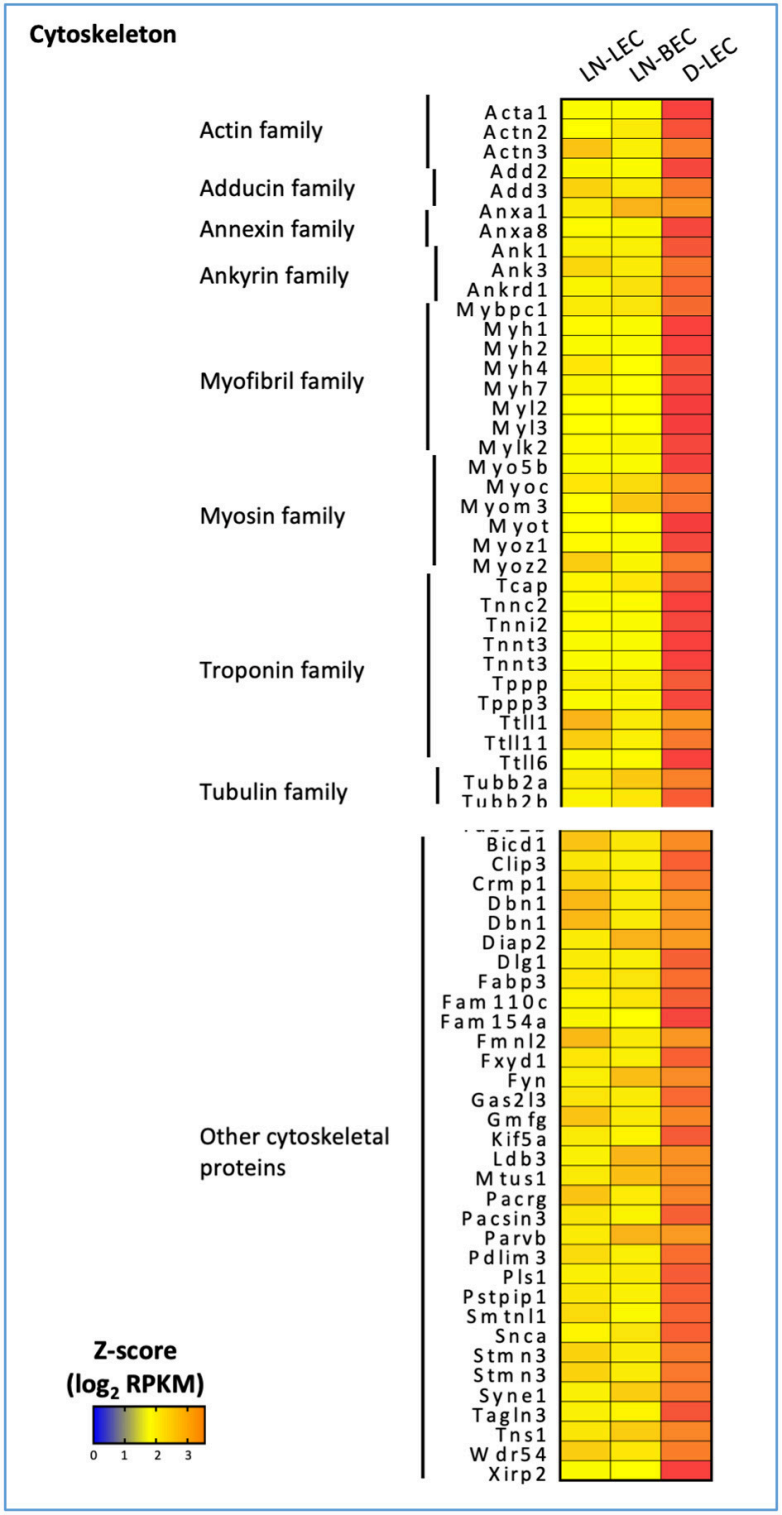
5X-DEG in D-LEC only subset showed enriched representation of different cytoskeletal protein groups compared to other 5X-DEG subsets. Heatmap analysis based on Z-score values of average log2 FPKM for replicates in each cell type (details in Methods).

Conversely, GO terms related to cytoskeleton were highly ranked in the D-LEC only 5X-DEG subset, and to a lesser extent, the LN-LEC+D-LEC subset. These subsets contained a variety of overexpressed genes encoding cytoskeletal proteins and binding molecules (Figure 3). The enhanced expression of these cytoskeletal proteins and binding molecules are likely attributes of LEC in peripheral tissue lymphatics that allow them to maintain shape in the face of ECM-mediated displacement during body movement.

### Chemokine Expression Patterns Suggest a Collaborative Division of Labor Between LEC and BEC in Maintaining Spatial Organization and Compartmentalization of Cells in LN

**Figure 4:**
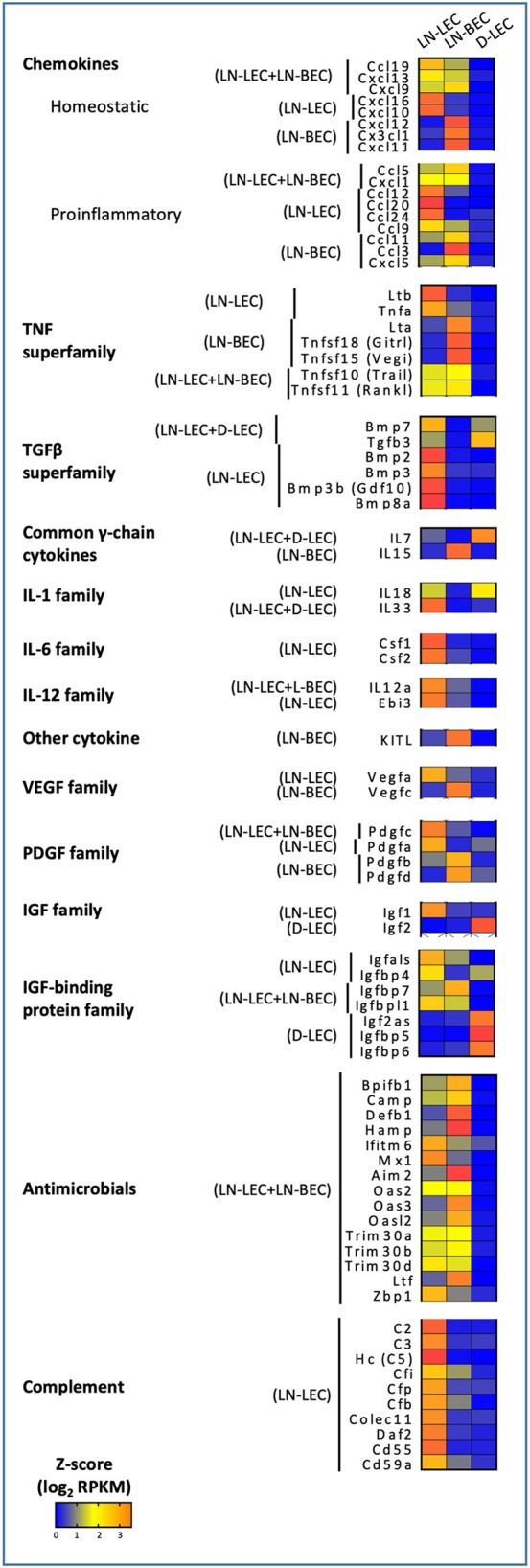
5X-DEG revealed immunomodulatory role of LEC and BEC via cytokines and innate effector molecules production. Heatmap analysis based on Z-score values of average log2 FPKM for replicates in each cell type (details in Methods).

GO terms related to chemokines were highly ranked in the LN-associated 5X-DEG subsets, but not those associated with D-LEC. Nonetheless, the patterns of chemokine expression in the LN-associated subsets revealed a surprising degree of complexity. The homeostatic chemokines CCL19 and CCL21 are two CCR7 ligands that have been implicated in homing of multiple immune cell subsets to LN via blood and lymph and organizing the T-cell zone of secondary lymphoid organs. CCL19 was overexpressed in the LN-LEC+LN-BEC subset (Figure 4), while CCL21 was not expressed at all in any analyzed EC population (<1 FPKM). While this is at odds with previous studies (10), we also confirmed using Q-PCR that CCL21 expression in LN-LEC was negligible compared to expression in bulk LN (Figure S3). Previous studies demonstrated the preferential ability of CCL19 to recruit CCR7^+^ cells compared to CCL21 (23–26) and that CCL19 signaling blocks directed migration of CCR7^+^ cells toward weak CCL21 signal (27). Our results suggest that autocrine secretion of CCL19 by LEC and BEC may play a role in organizing CCR7^+^ cells in the face of distinct gradients of CCL21.

The homeostatic chemokine CXCL13, a ligand for CXCR5 that organizes the B-cell zone of secondary lymphoid organs, was also overexpressed in the LN-LEC+LN-BEC subset (Figure 4). A previous study reported CXCL13 expression in LEC but not BEC isolated from peripheral LN (28), while another reported that it is highly expressed on HEV from Peyer's Patches (29). Because our LN samples were pooled from both peripheral and mesenteric LN, it is possible that LN-BEC in mesenteric LN may be similar to those in Peyer's Patches due to their close anatomical proximity. Nonetheless, insofar as CXCL13 appears to be essential for organization of LN but not B-cell entry (30, 31), our results suggest that both LN-LEC and LN-BEC have the potential to influence this process.

CXCL12, a chemokine that plays multiple roles but is particularly involved in homing of both T- and B-cells into LN (29, 32–34), was overexpressed only in LN-BEC (Figure 4). This is in keeping with its principle function in homing, as opposed to organization, of LN. LN-BEC also selectively expressed CX3CL1, a membrane associated chemokine that binds to CX3CR1^+^ cells. These include memory CD8 T-cells that reside in the LN-subcapsular cortex (35), and efferocytic T-zone macrophages (TZM) (36). There is no current evidence demonstrating that CX3CL1 mediates recruitment or localization of these cells in LN. To the contrary, CX3CR1 deficient TZM localize in normal numbers, but are deficient in clearance of apoptotic cells (36) because CX3CL1 also functions as an “eat me” signal (37) Nonetheless, the selective expression of CX3CL1 in LN-BEC is not entirely consistent with this role, and suggests that it may play a role in recruitment of cells to LN.

In contrast, LN-LEC overexpressed another membrane-bound chemokine CXCL16, which has activities as a chemoattractant and a scavenger receptor (Figure 4). As a chemoattractant it binds to CXCR6, which is expressed on activated CD8 and CD4 T cells (38–42). CXCL16 expressed in LN fibroblastic reticular cells was shown to mediate migration and mild adhesion of CXCR6^+^ CD8 and CD4 T cells (43). CXCL16 expression by LN-LEC could function similarly, and could mediate immune regulation of activated T cells along with other molecules such as PD-L1 and Lag3 (15, 16).

Perhaps surprisingly, the LN-LEC+LN-BEC, LN-LEC, and LN-BEC subsets each overexpressed several proinflammatory chemokines, which collectively support recruitment of a variety of immune cells, many of which are not resident in resting SLO (Figure 4). CXCL1 is essential for neutrophil migration and neutrophil extracellular trap formation (44), while CCL5 mediates recruitment of leukocytes expressing its cognate receptor, CCR5 (45).However, D-LEC do not overexpress any similar chemokines. Given the pervasiveness of blood and lymphatic vessels in the LN, the shared expression of these chemoattractant molecules seems consistent with a role in recruitment of T-cells and DC on the one hand and B-cells on the other. However, it is also conceivable that only well-localized subpopulations of each endothelial cell type express either chemokine, enabling them to participate in organizational processes.

CXCR3 is a chemokine receptor that is widely expressed on activated and memory type I CD4 and CD8 T cells. Interestingly, LN-LEC and LN-BEC both overexpress one CXCR3 ligand, CXCL9, while the two others, CXCL10 and CXCL11, are overexpressed only in LN-LEC or LN-BEC, respectively, although CXCL11 is a pseudogene in C57Bl/6 mice (Figure 4). CCR5 is a chemokine receptor with a similar expression pattern on T cells, and additionally on macrophages and dendritic cells, and two of its ligands, CCL5 and CCL3, are overexpressed by LN-LEC+LN-BEC and LN-LEC subsets, respectively (Figure 4). This suggests a subtle interplay between LN-LEC and LN-BEC in fine-tuning organization and movement of different antigen experienced and antigen presenting cells in the LN.

**Figure 5:**
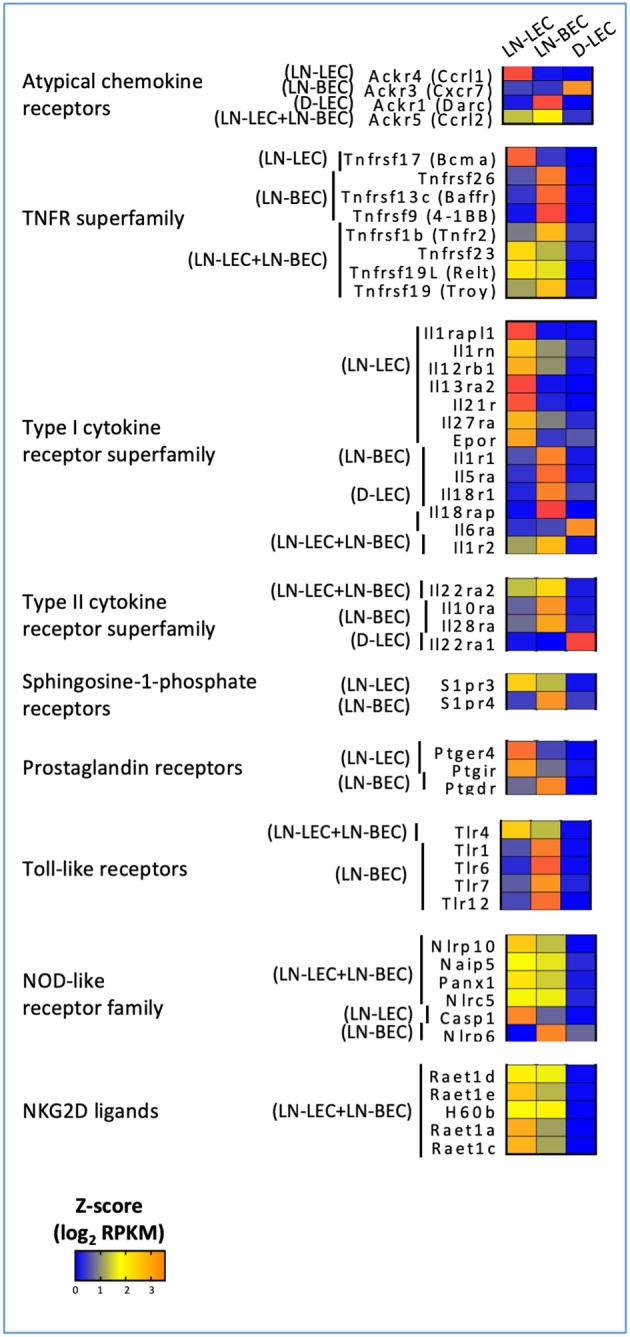
5X-DEG revealed immunosensory role LEC and BEC via cytokines and pathogen sensing. Heatmap analysis based on Z-score values of average log2 FPKM for replicates in each cell type (details in Methods).

In keeping with this idea, we also found overexpression of atypical chemokine receptors in LN-LEC, LN-BEC, D-LEC, and LN-LEC+LN-BEC subsets, but not the LN-LEC+D-LEC subset (Figure 5). These molecules typically function as decoy receptors to create chemoattractant gradients through chemokine sequestration. The LN-LEC+LN-BEC subset overexpressed ACKR5, which binds to CCL19. Since this same subset overexpresses CCL19, this may suggest that expression of the chemokine and the decoy receptor differs based on precise location within the LN. LN-LEC and D-LEC respectively overexpressed the atypical chemokine receptors, ACKR4 and ACKR3, which bind to CCL21, and CXCL11 and CXCL12, respectively. LN-BEC overexpressed ACKR1, which binds to CCL2, CCL5, CXCL1, CXCL4, CXCL5, CXCL7, and CXCL8. These data suggest that the expression of these chemokine decoy receptors may further augment cooperative interplay of LEC and BEC in controlling chemokine gradients in LN to promote directional sensing, migration, and activation of immune cells.

### TNF and TNFR Superfamily Expression Patterns Suggest Overlapping but Distinct Involvement of LEC and BEC in Maintenance of LN Microarchitecture and Involvement in Autocrine and Paracrine Signaling Mechanisms

GO terms related to TNF and TNFR superfamily members were also highly ranked in the LN-associated 5X-DEG subsets, but not those associated with D-LEC. The LN-BEC 5X-DEG subset overexpressed TNFR2 (TNFRSF1B) (Figure 5). TNFR1 (TNFRSF1A) and LTβR were overexpressed at similar levels in LN-LEC and LN-BEC relative to D-LEC, but the fold change was <5 (GSE119499). However, LN-LEC overexpressed TNFα and LTβ, while LN-BEC overexpressed LTα (Figure 4). Since LTα can form either a homotrimer that binds TNFRs or a heterotrimer with LTβ that binds LTβR, this suggests that these two populations differ in expression of these alternative forms, possibly leading to differences in autocrine or paracrine signaling that could influence lymphoid tissue microarchitecture. It is well-established that LTβR signaling is required form homeostatic maintenance of HEV phenotype, including expression of PNAd and MadCAM-1 (46) and one major source of LTα1β2 is dendritic cells (47). Conversely, LTβR signaling plays a role in expression of homeostatic chemokines (48) and dendritic cell maturation (49, 50). Our data suggest that dendritic cells influence LN-LEC and LN-BEC phenotypes via the LTβR signaling pathway, and vice versa. Additionally, the LN-LEC+LN-BEC 5X-DEG subset overexpressed RankL (Figure 4). The roles of these molecules in LN development and maintenance are well-established (51–56), but the specific involvement of endothelial cells as either initiators or recipients of TNF-related signaling has not been well-described.

Other overexpressed TNF and TNFR superfamily members have been associated with induction of cell death. The LN-LEC+LN-BEC subset overexpressed TRAIL (TNSFSF10), a TNF-superfamily ligand known to induce death of activated cells that express TRAIL receptors (57–60) (Figure 4). However, we did not detect pro-apoptotic TNFRSF10B (TRAIL-R2) expression in either LN endothelial subset. Instead, they expressed a decoy receptor for the ligand, TNFRSF23 (mDCTRAILR1) (61) (Figure 5). These data suggest that LEC and BEC in the LN induce TRAIL-mediated apoptosis of other cell types while protecting themselves. Conversely, the LN-LEC+LN-BEC 5X-DEG subset overexpressed TNFRSF19L (RELT). RELT has been shown to induce cellular cell death in multiple cell types via a mechanism distinct from TNFR1 (62). A ligand for RELT has not been identified (63). It is intriguing to consider whether RELT represents a mechanism by which inflammation driven LN angiogenesis and lymphangiogenesis might be downregulated after resolution.

Several overexpressed TNF and TNFR superfamily members have costimulatory or survival promoting functions. LN-LEC+LN-BEC overexpress TNFSF15 (VEGI, TL1A) (Figure 4), which acts as a T cell co-stimulator to induce a variety of distinct T cell subsets and immunopathologies (64–69) and promotes DC maturation (70). It also inhibits expression of VEGFR1 and induces endothelial apoptosis to inhibit vasculogenesis, but promotes lymphangiogenesis (71–74). LN-BEC selectively overexpress TNFSF18 (GITRL) (Figure 4), which co-stimulates both effector and regulatory T cells (75–77). Interestingly, LN-BEC also selectively overexpress TNFRSF9 (4-1BB), which could render them susceptible to signals delivered by 4-1BBL^+^ cells such as DC, and TNFRSF13C (BAFFR), which promotes B-cell survival and isotype switching (78, 79) (Figure 5). Another receptor for BAFF, TNFRSF17 (BCMA), which promotes survival of long-lived plasma cells (80) was overexpressed in LN-LEC (Figure 5). The impact of signals delivered by these receptors on endothelial function is unknown.

### Selective Expression of Multiple TGFβ-Superfamily Members Suggests That LN-LEC Contribute to Immunosuppressive Functions in Homeostasis

GO terms related to the TGFβ-superfamily were highly ranked in the LN-LEC+D-LEC and LN-LEC 5X-DEG subsets, but not in the D-LEC, LN-BEC, and LN-LEC+LN-BEC subsets. LN-LEC+D-LEC overexpressed TGFβ3 and BMP7, while LN-LEC overexpressed BMP2, BMP3, BMP3B, and BMP8A (Figure 4). TGFβ3 is highly homologous to TGFβ1 and TGFβ2, which were comparably expressed in all three endothelial populations. However, TGFβ3 binds more potently to TGFβ receptors I (TGFBR1/ALK-5) and II (TGFBR2) (81–83). TGFβ3 plays similar roles in immunosuppression and stimulation as TGFβ1 (84, 85), TGFβ3, BMP7, and BMP2 have been shown to suppress survival, proliferation, differentiation of *in vitro* grown human B cells into antibody-secreting cells (86–88). BMP2 inhibits T cell proliferation (89) and promotes Foxp3^+^ Treg generation in the context of TGFβ treatment (90). Little is known about the immunological activity of the other BMPs remaining molecules expressed in LN-LEC. Nonetheless, expression of these molecules may be associated with previously described tolerogenic properties of LN-LEC (13, 15, 16, 91).

### Expression of Cytokines and Innate Effector Molecules Suggests Additional Immunomodulatory Roles of LN-Localized LEC and BEC

GO terms for several additional cytokines and growth factors were highly ranked, almost exclusively in LN-associated 5X-DEG subsets. These included the common γ-chain cytokines, IL-7 and IL-15, which were overexpressed LN-LEC+D-LEC and LN-BEC, respectively (Figure 4). These observations corroborate earlier work (7), and suggest a division of labor between LEC and BEC in maintaining IL-7Rα^+^ and IL-15Rα^+^ cells in LN. They also point to D-LEC as a source of IL-7 for homeostatic T cell maintenance. LN-LEC+D-LEC also overexpressed IL-18, while LN-LEC expressed IL-33, both members of the IL-1 family. IL-18 synergizes with IL-7 in activation and priming of naïve CD8 T cells (92), in that IL-7 upregulates IL-18R. Finally, LN-LEC+LN-BEC and LN-LEC subsets overexpressed IL-12a (p35) and EBI3, respectively. IL-12a (p35) pairs with EBI3 to form IL-35 (93, 94), which has been demonstrated to induce inducible regulatory T cells (iTregs) (95), and suppress T cell proliferation (96). The LN-LEC+LN-BEC subset overexpressed KITL, a ligand for the cell surface tyrosine kinase KIT found on lymphocytes and hematopoietic stem cells, while LN-LEC overexpressed CSF1 and CSF2, ligands for CSFR1 and CSFR2 receptors expressed on macrophages. Collectively, these patterns of expression reinforce the expansive roles played by both LN endothelial populations, and particularly LN-LEC, in promoting the survival of different immune subpopulations, and in providing a context for their differentiation.

Consistent with earlier work (7), LN-LEC expressed the proangiogenic factor, VEGFA, while LN-BEC expressed the lymphangiogenic factor, VEGFC (Figure 4). This creates the possibility that these two cell types could cross-regulate one another. LEC and BEC also expressed several members of the PDGF family (Figure 4), which could support fibroblastic reticular cells. These data point to a dynamic co-dependence among different stromal cell types that may regulate the balance between cell populations under steady state and inflammatory conditions.

LN-LEC and D-LEC subsets, respectively overexpressed IGF1 and IGF2, distinct members of the insulin-like growth factor (IGF) family (Figure 4), while transcripts for IGF-family receptors (IGFR1 and IGFR2) were detected but not differentially expressed in all 3 endothelial populations. These 3 populations collectively overexpressed most of the IGF binding proteins (IGFBP), which bind to IGFs and modulate their activity in distinct ways (97–100). Together, these data demonstrated LEC in different anatomical niches control the local tissue milieus to support cellular growth, differentiation, and function via intricate networks of cellular IGF1- and IGF2-signaling and counter balance mechanisms to maintain tissue homeostasis.

GO terms associated with innate host defense mechanisms were also highly ranked in the LN-LEC+LN-BEC 5X-DEG subset, corresponding to a range of genes with antimicrobial activities to viral, bacterial, and fungal organisms (Figure 4). This suggests an as yet unappreciated role for LN endothelial populations to prevent pathogen dissemination. In addition, the LN-LEC 5X-DEG subset overexpressed a number of molecules associated with classical and non-classical complement cascades. These included C2, C3, and C5, which participate directly in the proteolytic cascade. The resulting products include the anaphylatoxins C3a and C5a, which serve as chemoattractant for neutrophils, monocytes, and macrophages (101–103), and modulate the functions of APCs and T cells (104–106), and C3b, which binds to pathogens, immune complexes, and apoptotic cells to promote phagocytosis (107). These data suggest that LN-LEC may collaborate with subcapsular sinus macrophages, follicular dendritic cells, and B cells to promote both innate and adaptive immune responses through complement component secretion. LN-LEC also overexpressed of CD55 (DAF), DAF2, and CD59a, all of which prevent formation of the membrane attack complex and enable LN-LEC to protect themselves from the actions of the products they secrete.

### Expression of Cytokine Receptors and Pathogen Sensing Molecules Suggests Additional Immunosensory Roles of LN-Localized LEC and BEC

GO terms for several cytokine receptors and pathogen sensing molecules were highly ranked, again almost exclusively in LN-associated 5X-DEG subsets. LN-LEC+LN-BEC, LN-BEC, and most prominently, LN-LEC, overexpressed members of the type I cytokine receptor superfamily, including components of the IL-1, IL12, IL18, IL27 receptors, and antagonists and decoys (Figure 5). LN-LEC and LN-BEC also overexpressed receptors for several other immune relevant molecules, including IL10, sphingosine-1-phosphate, and prostaglandins. These data indicate that the LN endothelial cells are poised to sense and respond to a variety of cytokine cues in their local milieu, although the consequences of signaling by any given receptor remain to be established.

LN-LEC, LN-BEC, and LN-LEC+LN-BEC overexpressed several toll-like receptors (TLR) and NOD-like receptors (NLR). LN-LEC+LN-BEC overexpressed TLR4, while several other TLR were selectively overexpressed by LN-BEC. The patterns of NLR overexpression were more complex. As with the immune receptors above the consequences of signaling by any given TLR or NLR remain to be established. Interestingly, however, LN-BEC overexpressed NLRP6, which inhibits inflammasome formation (108, 109). The LN-LEC+LN-BEC 5X-DEG subset overexpressed several ligands for the NKG2D receptor that is expressed on NK, NKT, γδ T cells, and activated CD8 T cells (Figure 5). While these ligands are generally associated with promoting effector activity via NKG2D signaling, RAET1E expressed on endothelial cells was demonstrated to inhibit NK cell activation by inducing NKG2D internalization (110). This raises the question of whether the responses of LN-LEC and LN-BEC to prototypical pro-immune receptor signaling may be counter-regulatory.

### LN-LEC and LN-BEC Overexpress Molecules Involved in MHC-I and MHC-II Antigen Processing and Presentation

**Figure 6:**
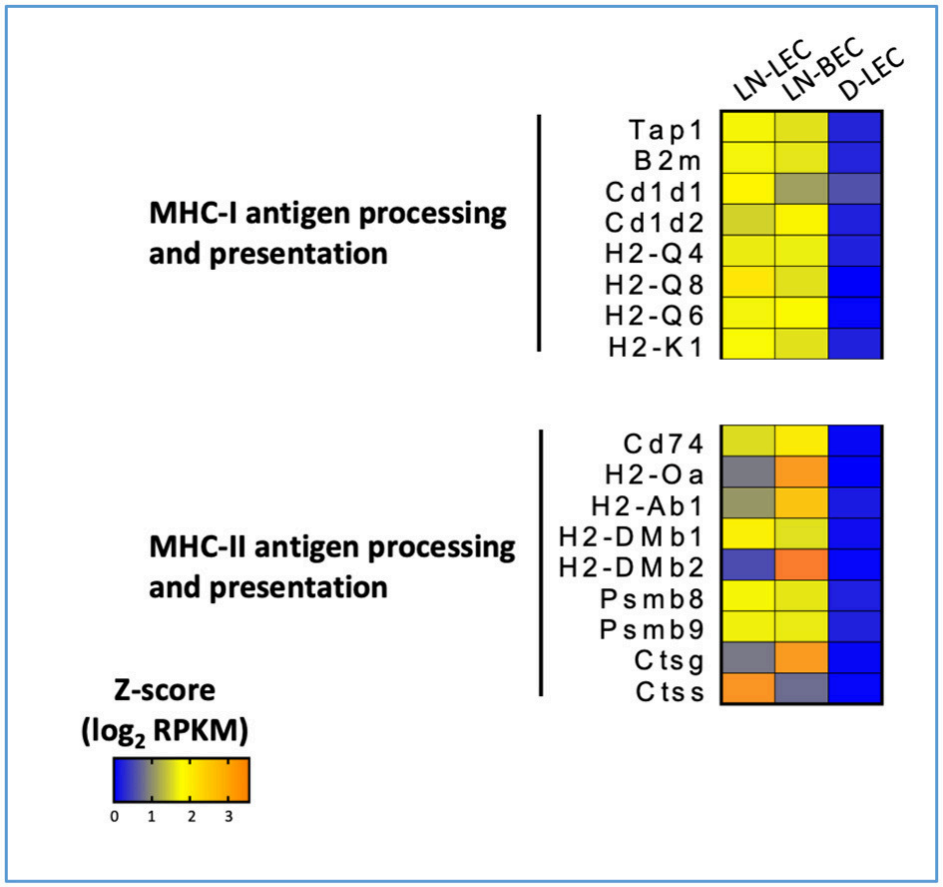
5X-DEG in LN-LEC+LN-LBEC shared subset revealed overrepresentation of molecules involved in antigen processing and presentation via MHC-I and MHC-II pathways. Heatmap analysis based on Z-score values of average log2 FPKM for replicates in each cell type (details in Methods).

GO terms for MHC-I and MHC-II antigen processing and presentation pathways were highly ranked exclusively in the LN-LEC+LN-BEC 5X-DEG subset. Overexpressed MHC-I pathway components included H-2K^b^, β2 microglobulin, several Qa and Cd1d molecules, TAP1 and 2 components of the immunoproteasome (Figure 6). Overexpressed MHC-II pathway components included H-2Aβ, invariant chain, peptide editors (H-2Oα, H-2DMβ2, H-2DMβ1), and cathepsins S and G. While not achieving 5X differential expression, the MHC-I H-2D^b^ molecule, and the MHC-II components H-2Aα and H-2DMα were also overexpressed in the two LN cell populations relative to D-LEC (GSE119499). Cathepsin L was also overexpressed by 5X in LN-LEC relative to D-LEC, but this did not give rise to an enriched GO term score (Table S1). These data reinforce previous studies from our lab demonstrating that LN-LEC efficiently present endogenous antigens via H-2K^b^ to CD8 T cells (14–16), and suggest that LN-LEC have elevated capacity to present antigens via additional classical and non-classical MHC-I molecules. They also suggest that LN-BEC have a similar capacity. We have also reported that LN-LEC are unable to present endogenous or exogenous antigens to CD4 T cells despite expressing MHC-II molecules, and have suggested that this is due to a deficiency in H-2DM expression (16). While both H-2DMα and H-2DMβ were overexpressed in LN-LEC, their level of expression was still low (<100 FPKM), compared with FPKM values >1000 for H-2Aα, H-2Aβ, invariant chain, and cathepsins S and L, consistent with this earlier conclusion. We have concluded that the MHC-II molecules expressed on LN-LEC are primarily involved in engaging LAG-3 on T cells to induce peripheral tolerance (16). Given the similar expression of MHC-II components in LN-LEC and LN-BEC, we suggest that the latter cells serve a similar function.

### LN-LEC Express Elevated Number of Molecules Involved in Exogenous Material Acquisition That Potentially Contribute to Their Functions in Antigen Archival and Peripheral Tolerance

**Figure 7:**
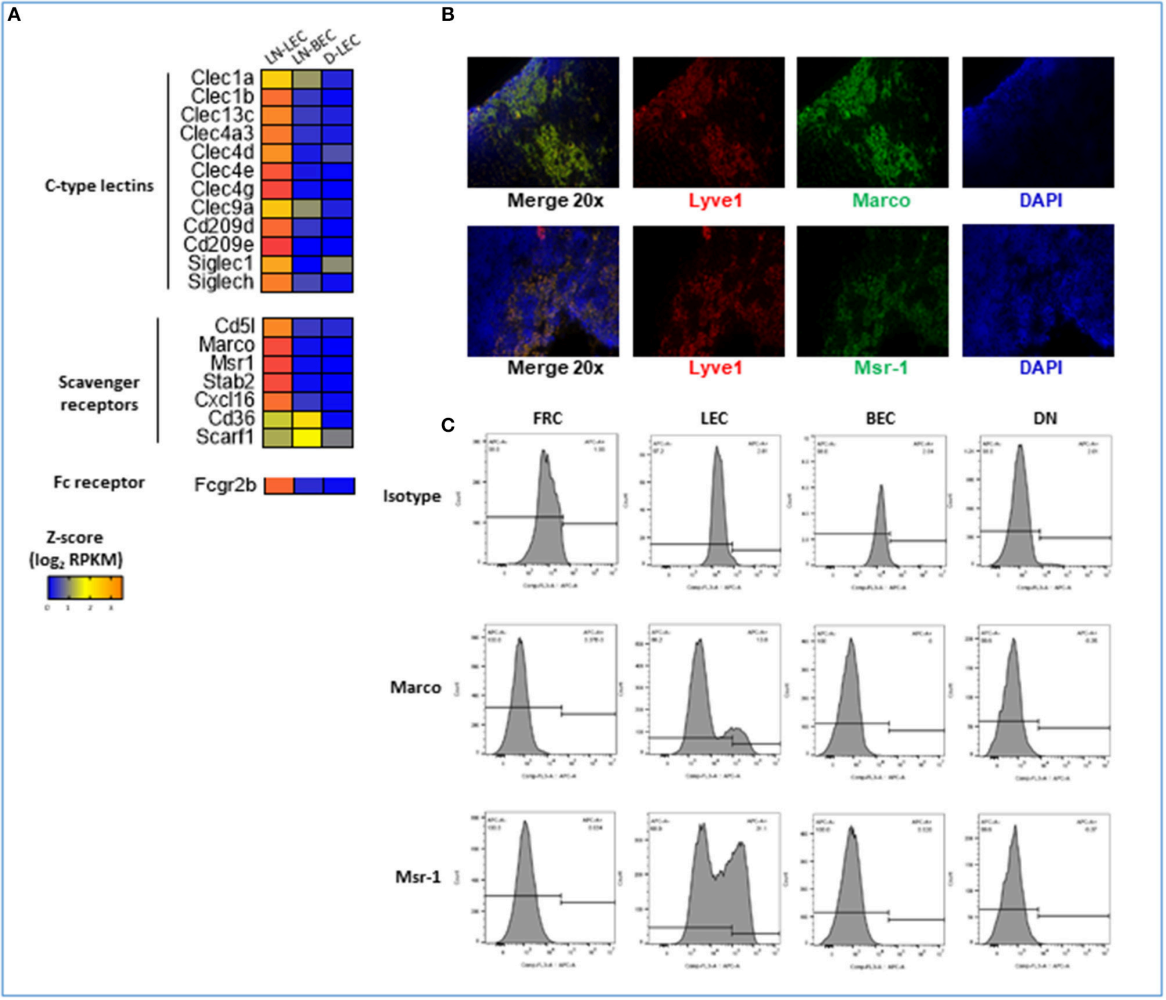
5X-DEG in LN-LEC only subset revealed enriched overrepresentation of molecules involved in receptor-mediated endocytosis and scavenger receptor activity. **(A)** Heatmap analysis based on Z-score values of average log2 FPKM for replicates in each cell type (details in Methods). **(B)** Flow cytometry analyses of LN stromal cell populations for expression of Marco and Msr-1. **(C)** Immunofluorescent (IF) staining in adjacent tissue sections of LN for detection of Marco and Msr-1 co-expression with Lyve-1 (LEC marker).

GO terms for receptor mediated endocytosis were highly ranked almost exclusively in the LN-LEC 5X-DEG subset. Overexpressed molecules included C-type lectin receptors, scavenger receptors, and Fc receptors (Figure 7A). C-type lectin receptors have been categorized as binding to either carbohydrate, non-carbohydrate structures, or both. LN-LEC overexpressed some C-type lectins that bind carbohydrates (CLEC4A3, CLEC4D, CLEC4E, CLEC4G, CD209D) and others that bind non-carbohydrates (CLEC1A, CLEC1B, CLEC9A). We found a single C-type lectin (CLEC4G) in the LN-LEC+LN-BEC, although this subset was not enriched for GO terms associated with receptor mediated endocytosis. C-type lectin receptors also can signal via immune tyrosine activation or inhibitory motifs, or through non-canonical structural features that mediate positive or negative immune stimuli. C-type lectin receptors in the LN-LEC include all 3 of these signaling categories.

The scavenger receptors MSR-1 (SR-A1), MARCO (SR-A6), STAB-2 (SR-H2), and CXCL16 (SR-G1) were also overexpressed in LN-LEC (Figure 7A). Previous studies demonstrated that the class A scavenger receptors, MSR-1 and MARCO, are expressed primarily on macrophage subpopulations, and are associated with recognition of surface molecules of Gram-positive and -negative bacteria (111, 112), modified and oxidized LDL (113, 114), hepatitis C virus (115), β-amyloid (116), and heat shock proteins (117). The expression of MSR-1 and MARCO under steady state condition was reported to be restricted to macrophages in the LN and the marginal zones of the spleen (118). Our data extends this. In addition, we demonstrated by immunofluorescence and flow cytometry that subpopulations of LN-LEC express MARCO and MSR-1, while LN-BEC, fibroblastic reticular cells (FRC), and other CD45^neg^ LN stromal cell populations do not (Figures 7B,C). Membrane-bound CXCL16 and STAB-2 bind phosphatidylserine and oxidized lipids (119–123), and membrane-bound CXCL16 also mediates phagocytosis of bacteria (124). LN-LEC also overexpressed the Fc receptor, FCGR2B, which has been shown to be essential for internalization of immune complexes by DC (125–127).

Again, while the subset did not have high GO enrichment scores, LN-LEC+LN-BEC also expressed two scavenger receptors, CD36 (SR-B2) and SCARF-1 (SR-F1). SCARF-1 and CD36 have been previously reported as pattern recognition molecules for fungal pathogens in innate immunity (128). More recently SCARF-1 expressed by splenic DC, macrophages, and endothelial cell was shown to bind the complement molecule, C1q to mediate apoptotic cell clearance, thus preventing generation of autoantibodies to DNA-containing antigens that lead to lupus-like disease and autoimmunity (129). CD36 was reported to facilitate transfer of surface antigen between CD8α^+^ DC and mTEC to promote tolerance to self-antigens during T cell development (130).

Collectively, these data point to a previously undescribed but comprehensive capability of LN-LEC to internalize a broad array of extracellular materials using a variety of pattern recognition elements, either alone or in conjunction with other immune recognition molecules. We suggest that many of these material may be delivered into antigen processing and presentation pathways, at least for MHC-I molecules (13, 16). Our previous work indicates that the MHC-II pathway is non-functional in LN-LEC in the steady state (16), but it remains possible that this changes under conditions of pathogen exposure and inflammation. An important question is whether this is primarily a means of generating tolerance to exogenous self-antigens, or whether LN-LEC may sometimes also serve as accessory antigen presenting cells during an active immune response. In addition, this internalization capability is likely to be important in the antigen acquisition and archiving functions of LN-LEC (17, 18).

### Attempting to Identify Peripheral Tissue Antigens Expressed by LN-LEC

We previously demonstrated that LN-LEC adventitiously expressed transcripts for proteins otherwise restricted to a small number of non-hematopoietic, non-endothelial peripheral tissues, typified by Tyr (13, 14). Because expression of these molecules is not dependent on Aire, as is the case for medullary thymic epithelial cells (131), it has not been possible to characterize the full range of peripheral tissue antigens potentially displayed by LN-LEC. With the expansion of available data on tissue specific expression, it has also become more difficult to unambiguously identify genes whose expression is rigorously limited to only a small number of tissues. Using the characteristics of Tyr expression (FPKM <343), we hypothesized that there would be an elevated number of transcripts at or below this expression level in LN-LEC compared to D-LEC. However, we found that these two subsets contained equal number of 5X-DEG that met this criterion (Table S4). Thus, although the cut-off criteria based on Tyr expression is a good first step, a more comprehensive approach is needed to identify the candidate peripheral tissues antigens expressed by LN-LEC.

### Conclusions

This study provides comprehensive comparative transcriptomic analyses of LN-LEC, LN-BEC, and D-LEC and has defined in detail gene expression differences that point to functional specializations of EC in different anatomical locations. Our goal was to provide a broad compendium of gene expression differences based on anatomic location and endothelial lineage, with a focus on genes of immunological interest. We believe that this information will be a significant and extremely useful resource for many workers in this field. Our data identify significantly expanded cohorts of immunologically significant genes either shared by LN-localized ECs, or expressed distinctly by one or the other LN-localized subset. These genes extend the understanding of both populations as regulators, not only of hematopoetic cell trafficking, but also cellular differentiation. They also point to an emerging understanding that LN-localized EC express a variety of receptors that enable them to sense immunologically relevant changes in their environment. Further exploration of the consequences of this sensing on EC proliferation, differentiation, and function in immunoregulation is an important area for further work. This study clarifies that both LN-localized EC function as antigen presenting cells, and this issue explored in somewhat greater detail elsewhere (Santambrogio et al., Manuscript Submitted^1^. Finally, this study highlights the surprising number of molecules involved in uptake of exogenous materials expressed distinctly by LN-LEC. The involvement of these molecules in enabling antigen archiving and peripheral tolerance to exogenous self- antigens is another rich area for further exploration.

While our comparative analysis identifies profound differences in bulk populations that are based on anatomical location, it does not address the almost certain heterogeneity that exists at the single cell level in each location, some of which has been pointed to in our own previous work (14). However, given the limited depth of single cell coverage, many of the differences we have identified might not have been immediately evident with that approach. Nonetheless, the results presented here provide a springboard for further work to establish the existence of heterogeneity in expression within LN anatomical niches using a variety of technical approaches. Taken together, the comparative gene expression profiles provided here would be useful resources for future work to uncover novel mechanisms of endothelial functionality and specialization in peripheral tissues and LN.

## Materials and Methods

### Mice

C57BL/6 mice (6–8 weeks of age) were purchased from NCI and were housed at pathogen-free facilities at the University of Virginia. All procedures were carried out in accordance with the recommendations in the Guide for the Care and Use of Laboratory Animals of the National Institutes of Health and were approved by the University of Virginia Animal Care and Use Committee.

### LEC and BEC Isolation and Cell Sorting

Inguinal, axillary, brachial, cervical, and mesenteric LN were harvested, pooled, mechanically disrupted, and enzymatically digested for 15 min, followed by MACS bead depletion of CD45^+^ cells as previously described (16). Diaphragm tissues were treated in the same way. CD45^neg^ cells were electronically sorted based on absence of expression of CD45 (eBioscience, clone 30-F11), expression of pan-endothelial marker, CD31 (eBioscience, clone 390), and presence or absence of PDPN (Biolegend, clone 8.1.1) to distinguish LEC from BEC. Cells were collected in RNA Protect (Qiagen).

### RNA Extraction, cDNA Library Construction, and Sequencing

Total RNA was purified using RNAeasy mini kit (Qiagen) per manufacturer's instructions. cDNA library preparation and sequencing were performed by the Genomic Services Laboratory at Hudson Alpha, USA. Briefly, purified total RNAs (RIN score of 7.0 or higher) were prepared for sequencing using the Ovation RNA-Seq System V2 kit (Nugen) followed by RNA-Seq of 100 paired-end reads using the Illumina HiSeq 2500 v4 platform.

### Mapping, Quantification, and Differential Gene Analysis

Raw RNA-Seq read quality was assessed using FastQC (132) and low-quality regions were trimmed using Fastx-trimmer (http://hannonlab.cshl.edu/fastx_toolkit/index.html). Cleaned reads were aligned to the mouse reference genome (build mm9) using STAR (133) and read counts on known mouse genes were calculated using featureCounts, part of the Subread package (134). Next, uniquely aligned reads were analyzed using the DEseq2 package in the R statistical computing environment (R Development Core Team, 2011, http://www.R-project.org/) to obtain normalized counts, estimate dispersion, and determine a negative binomial model for each gene. Principal Component Analysis was performed on rlog-transformed counts for quality assessment. Differentially expressed genes (DEG) were determined using DESeq2 and the Benjamini-Hochberg False Discovery Rate procedure was used to re-estimate the adjusted *p*-values. In our analyses, DEG were identified as those with an average FPKM of 1 or greater and replicate comparisons of *p*-adjusted < 0.05 in all cell types. 5X-DEG were identified as those with fold-change of 5 or greater. Hierarchical gene clustering analysis was performed using complete linkage and Euclidean distance as measure of similarity to display the DEG expression patterns.

### Gene Ontology Analysis of 5X-DEG Subsets

Gene ontology analysis was performed with GOrilla software using the two ranked lists method (135). We used the 5X-DEG subsets as the target set and the all annotated genes from mouse reference genome (build mm9) as the background set. Briefly, GOrilla generates an enrichment score, which is the number of genes in the intersection of genes in the GO term (designated as B) and the number of genes in the target set (designated as b) for each associated GO term. We used the list of 5X-DEG for each subset as target. Enrichment of GO terms is then tested for statistical significance using a hypergeometric test and *p*-adjusted < 0.001 was considered as significant. Analyses were performed against gene ontologies: biological process and molecular function. We then identified the relationship between GO terms using hierarchical directed acyclic graph generated by GOrilla.

### Immunofluorescence Staining and Flow Cytometry Analyses of LN-LEC

Immunofluorescence staining of adjacent LN tissue sections of C57BL/6 mice (purchased from NCI) were performed using rat anti-mouse LYVE-1 (R&D Systems, MAB2125), goat anti-mouse MARCO (R&D Systems, AF2956), goat anti-mouse MSR1 (R&D Systems, AF1797), normal goat IgG (R&D Systems, AB-108-C), and rat IgG2a (R&D Systems, MAB006) antibodies at final concentrations of 5 μg, respectively. Subsequent detection were performed using donkey anti-goat IgG-FITC conjugated (R&D Systems, F0109) and mouse anti-rat IgG2a (eBioscience, cat# 11-4817-82) at manufacturers' recommended concentrations. Flow cytometry detection of MARCO and MSR1 were performed using cells gated on CD31 and gp38 expressions as described above using anti-mouse MARCO-APC conjugated (R&D Systems, FAB 2956A), anti-mouse MSR1-APC conjugated (R&D Systems, FAB1797A), and isotype control antibodies (R&D Systems, IC005A and IC006A).

## Data Availability

The RNA-Seq datasets generated from this study have been deposited in GEO archives under the accession number GSE119499.

## Ethics Statement

This study was carried out in strict accordance with the recommendations in the Guide for the Care and Use of Laboratory Animals of the National Institutes of Health. Procedures were approved by the University of Virginia Animal Care and Use Committee.

## Author Contributions

SR, AW, JP, and KC purified the cells and extracted the RNA. AR performed the qRT-PCR. SB performed immunofluorescence staining and flow cytometry analyses. AK and ST performed bioinformatics and statistical analyses. SB and NG analyzed the data. SB, AW, and VE designed the figures. SB and VE wrote the manuscript.

### Conflict of Interest Statement

The authors declare that the research was conducted in the absence of any commercial or financial relationships that could be construed as a potential conflict of interest.

## Footnotes

^1^Santambrogio L, Berendam SJ, Engelhard VH. The antigen processing and presentation machinery in lymphatic endothelial cells. *Front Immunol*. In Review.

## Abbreviations

BEC: blood endothelial cells
LEC: lymphatic endothelial cells
LN: lymph node
LN-BEC: lymph node-associated blood endothelial cells
LN-LEC: lymph node-associated lymphatic endothelial cells
D-LEC: lymphatic endothelial cells from diaphragm
DC: dendritic cells
DEG: genes differentially expressed between any two cell types
5X-DEG: genes whose differential expression in pairwise comparisons was greater than 5-fold
GO: Gene Ontology
ECM: extracellular matrix.
