# Supplementary material for: Comparative Transcriptomic Analysis Identifies a Range of Immunologically Related Functional Elaborations of Lymph Node Associated Lymphatic and Blood Endothelial Cells
Source: Front Immunol. 2019 Apr 16;10:816. doi: 10.3389/fimmu.2019.00816 (PMC6478037; doi:10.3389/fimmu.2019.00816)
Supplement: Supplementary file 5 [file Data_Sheet_1.PDF]

(a)

Female C57BL/6 (6-8 weeks)

Lymph node | **Diaphragm**

Enzymatic digestion

Single cell suspension

$CD45^+$ MACS depletion

Electronic cell sorting as in (b):

$CD45^{neg}$, $CD31^+$, PDPN+ or neg

RNA extraction

RNA-sequencing

Data validation

Transcriptome analyses

(b) **Gating strategy for LN-LEC and LN-BEC:**

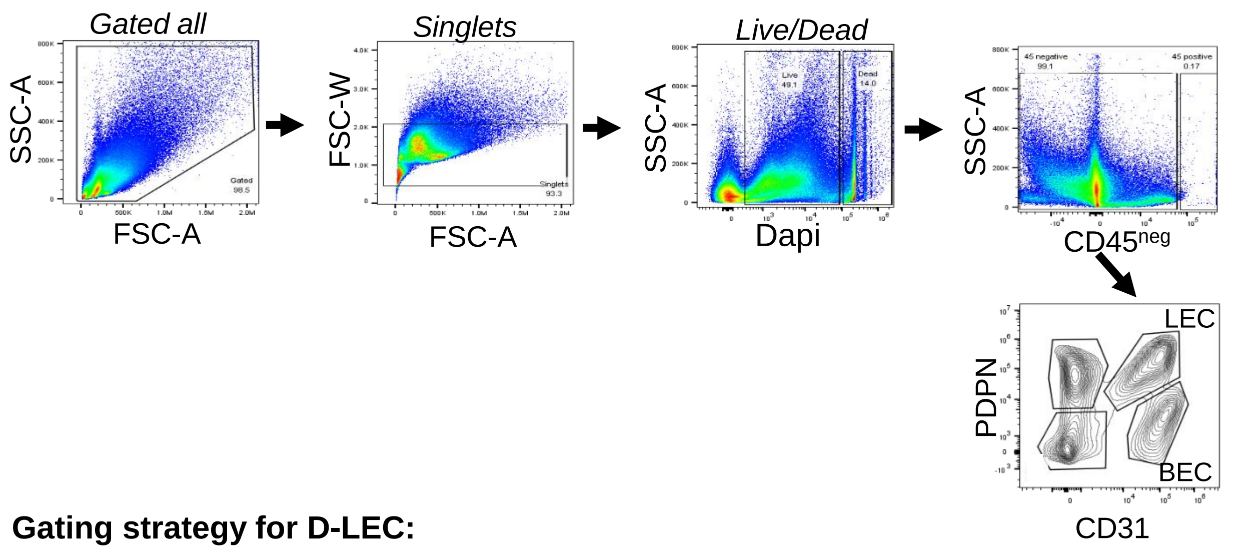

Gating strategy for D-LEC:

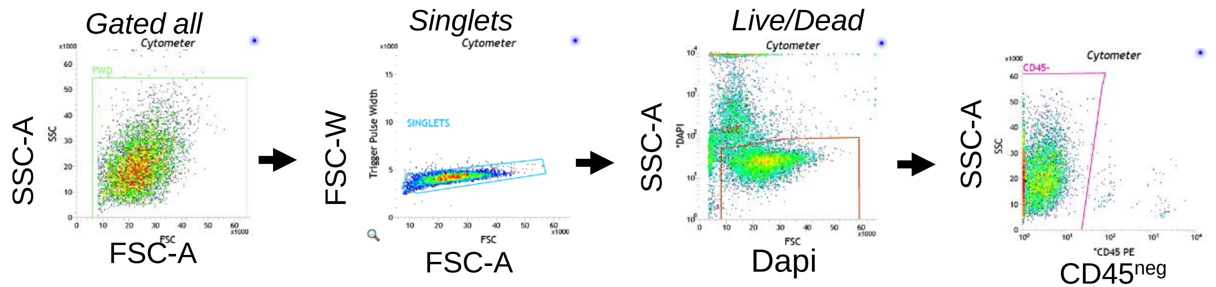

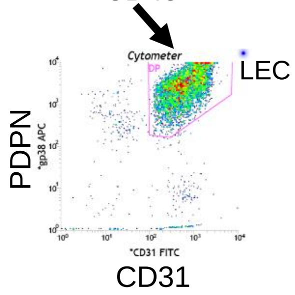

(c)

Population	Average purity	Expressed genes (Average FPKM>1)
LN-LEC (4 replicate samples)	98.62%	14,718
LN-BEC (3 replicate samples)	95.33%	14,893
D-LEC (3 replicate samples)	92.65%	14,384

Figure S1

Figure S1. (A) Schematic of cell isolation method of LEC and BEC from LN and diaphragm. (B) Flow analysis workflow and gating strategy for sorting LEC and BEC from LN (top row) and diaphragm (bottom row). (C) Percentage of post-sorting purity based on live cells for LN-LEC, LN-BEC, and D-LEC.

(a)

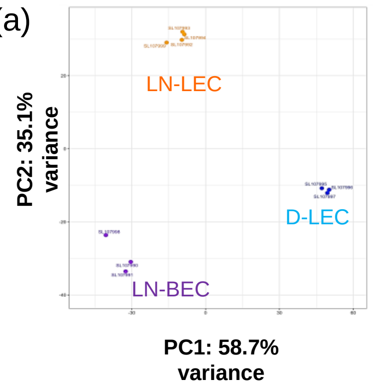

(b)

5X-DEG subset	Biological process GO (p-adj < 0.001)	Number of 5X-DEG associated	Molecular function GO (p-adj < 0.001)	Number of 5X-DEG associated
LN-LEC	391	463	61	622
LN-BEC	443	630	50	602
D-LEC	131	417	44	483
LN-LEC+LN-BEC	429	442	61	456
LN-LEC+D-LEC	59	137	7	105
D-LEC+LN-BEC	0	0	0	0

(c)

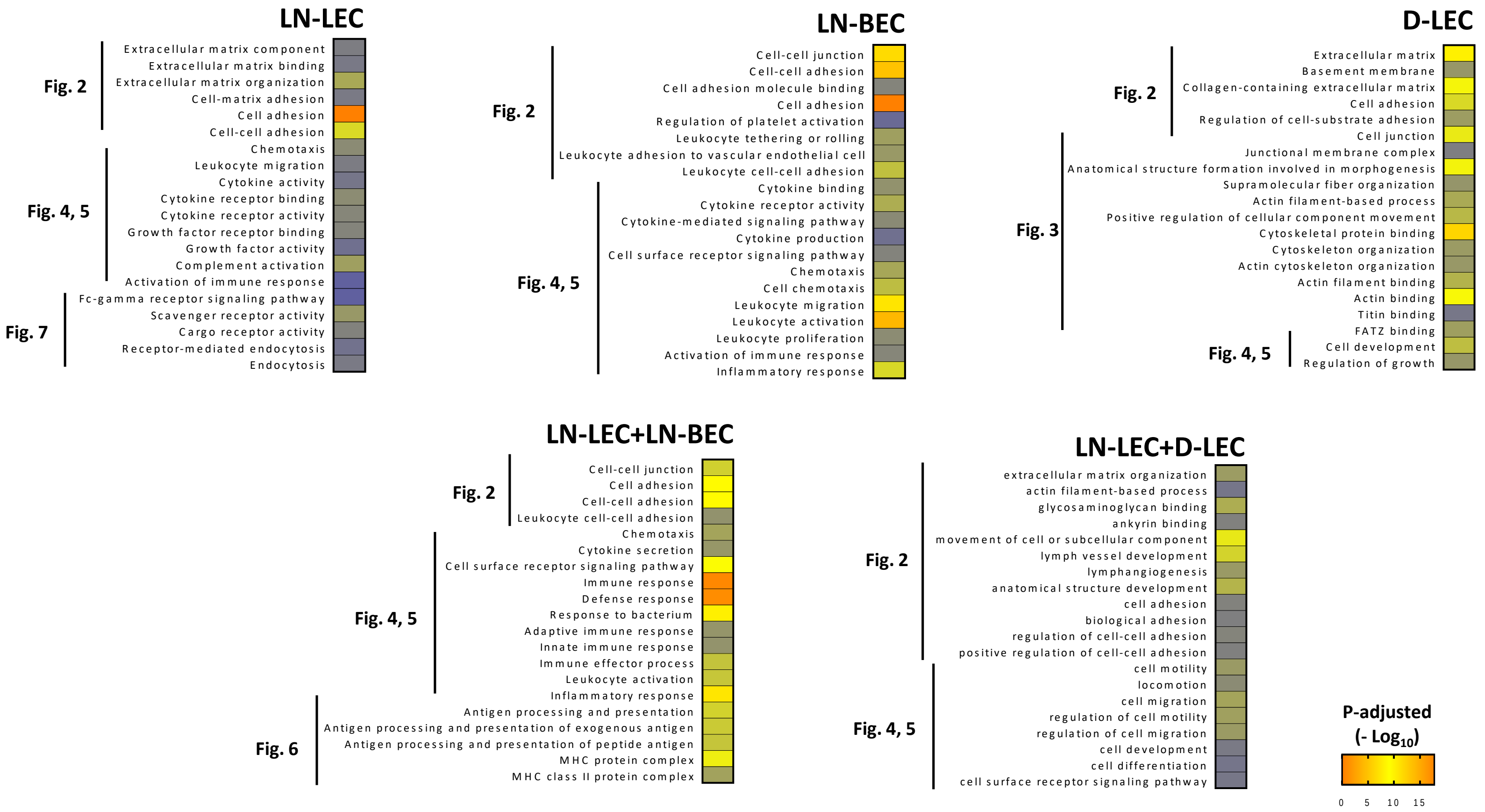

Figure S2

Figure S2. (A) Principal component analysis of expressed genes (average FPKM of 1 or greater) in LN-LEC, LN-BEC, and D-LEC. (B) Summary of gene ontology (GO) analyses in 5X-DEG subsets. (C) Top 20 GO terms showing significant enrichment (p-adj < 0.001, enrichment score > 2) in all five 5X-DEG subsets.

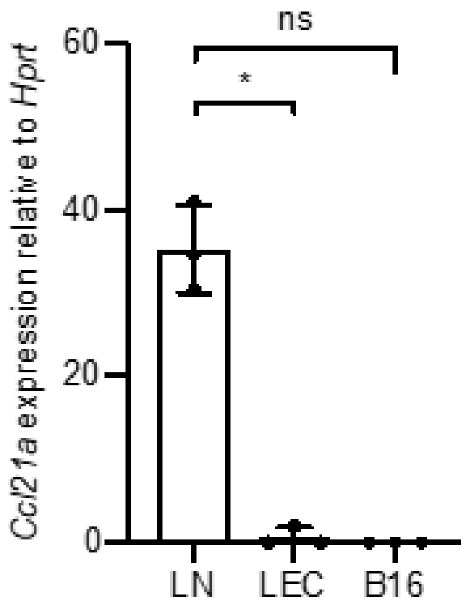

Figure S3. Lymphatic endothelial cells express significantly low levels of homeostatic chemokine CCL21. Expression of *Ccl21a* mRNA from whole lymph node lysates (n = 3), sorted lymphatic endothelial cells (n = 3) and cultured B16-F1 (n = 3) cells was determined by quantitative PCR. Data is presented as 2^{-DCt} relative to *Hprt*. Values shown are mean ± s.d. ns: $P>0.05$ and $*P<0.05$. P-values were calculated using a Kruskal-Wallis test with Dunn's posttest.

Figure S3
